# Caspase-4 Has Potential Utility as a Colorectal Tissue Biomarker for Dysplasia and Early-Stage Cancer

**DOI:** 10.1016/j.gastha.2024.09.007

**Published:** 2024-09-16

**Authors:** Laura E. Kane, Brian Flood, Joan Manils, Donna E. McSkeane, Aoife P. Smith, Miriam Tosetto, Fatema Alalawi, Joanna Fay, Elaine Kay, Cara Dunne, Stephen McQuaid, Maurice B. Loughrey, Jacintha O’Sullivan, Elizabeth J. Ryan, Kieran Sheahan, Glen A. Doherty, Emma M. Creagh

**Affiliations:** 1 School of Biochemistry and Immunology, Trinity Biomedical Sciences Institute, Trinity College Dublin, Dublin, Ireland; 2 Serra Húnter Programme, Immunology Unit, Department of Pathology and Experimental Therapy, School of Medicine, Universitat de Barcelona, L'Hospitalet de Llobregat, Spain; 3 School of Biological Sciences, Technological University Dublin, Dublin, Ireland; 4 Centre for Colorectal Disease, St Vincent’s University Hospital and School of Medicine and Medical Sciences, University College Dublin, Dublin, Ireland; 5 Pathology Department, Royal College of Surgeons Ireland and Beaumont Hospital, Dublin, Ireland; 6 Department of Surgery, Trinity St. James’s Cancer Institute and Trinity Translational Medicine Institute, St. James’s Hospital and Trinity College Dublin, Dublin, Ireland; 7 Precision Medicine Centre of Excellence, Patrick G Johnston Centre for Cancer Research, Queen’s University Belfast, Belfast, UK; 8 Department of Cellular Pathology, Royal Victoria Hospital, Belfast Health and Social Care Trust, Belfast, UK; 9 Centre for Public Health, Queen’s University Belfast, Belfast, UK; 10 Department of Biological Sciences, Limerick Digital Cancer Research Centre, Health Research Institute, University of Limerick, Limerick, Ireland; 11 Department of Pathology, St. Vincent’s University Hospital, Dublin, Ireland

**Keywords:** Colorectal Cancer, Caspase-4, Inflammatory Bowel Disease, Colonic Polyp, Biomarker

## Abstract

**Background and Aims:**

Colorectal cancer (CRC) is the second most deadly cancer globally. The rapidly rising incidence rate of CRC, coupled with increased diagnoses in individuals <50 years, indicates that early detection of CRC, and those at an increased risk of CRC development, is paramount to improve the survival rates of these patients. Here, we profile caspase-4 expression across 2 distinct CRC development pathways, sporadic CRC (sCRC) and inflammatory bowel disease-associated CRC (IBD-CRC), to examine its utility as a novel biomarker for CRC risk and diagnosis.

**Methods:**

Tissue samples from patients with CRC, colonic polyps, IBD-CRC, and sCRC were assessed by immunohistochemistry for caspase-4 expression in epithelial and stromal compartments. RNAseq expression data for caspase-4 in CRC and normal tissue samples were mined from online databases.

**Results:**

Epithelial caspase-4 expression is selectively elevated in CRC tumor tissue compared to adjacent normal tissue, where it is not expressed. In the sCRC pathway, caspase-4 is expressed in the epithelial and stromal tissue of all histological subtypes of colonic polyps, with a significant increase in epithelial expression from low-grade dysplasia to high-grade dysplasia progression. For the IBD-CRC pathway, caspase-4 epithelial expression was specifically upregulated in dysplastic and neoplastic tissue of IBD-CRC but was not expressed in normal or inflamed tissue.

**Conclusion:**

This study demonstrates that epithelial caspase-4 is selectively expressed in colon tissue during the development of dysplasia. As such, epithelial caspase-4 represents a promising novel tissue biomarker for CRC risk and diagnosis.

## Graphical abstract

**Figure:**
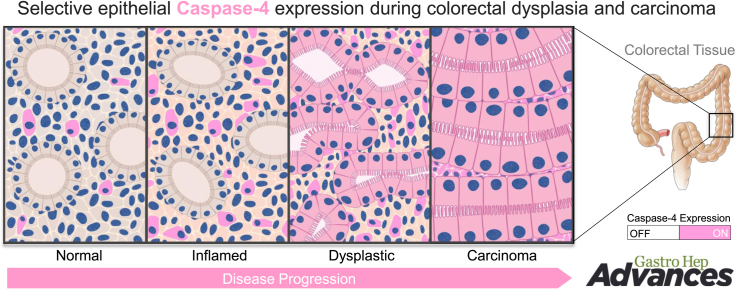

## Introduction

Colorectal cancer (CRC) is the second leading cause of cancer-related death in the United States, surpassed only by lung cancer.^1^ Roughly 7% of CRC deaths are estimated to be in individuals younger than 50 years.^2^ With CRC-related deaths in individuals <55 years rising by 1%–2% per year, increasing mortality among young adults represents a major challenge of health-care systems globally.^1^ Indeed, current statistical trends indicate that CRC is rapidly shifting to diagnosis at a younger age, and at a more advanced stage.^2^ Early detection of those at an increased risk of developing CRC, and of individuals with early-stage CRC, is key to improving survival rates in this cancer.

CRC development can occur via 2 distinct pathways: sporadically through the generation of a precancerous polyp; or following the long-standing intestinal inflammation associated with inflammatory bowel diseases (IBDs).^3^^,^^4^ Most CRCs are sporadic (sCRC) and arise via the adenoma-carcinoma sequence, originating through the development of an adenomatous polyp; the more recently described serrated pathway, involving sessile serrated lesions, having been shown to account for 15%–30% of CRCs.^5^^,^^6^ While IBD-associated CRC (IBD-CRC), developing through the inflammation-dysplasia-carcinoma sequence, is responsible for just 1%–2% of all CRC cases, this pathway accounts for a disproportionate number of CRC-related deaths.^7^, ^8^, ^9^ Indeed, IBD-CRCs have been demonstrated to occur in younger individuals and have worse outcomes overall compared to sCRC.^4^^,^^7^^,^^8^ As such, while IBD-CRC accounts for only a small proportion of CRC cases overall, patients with IBD are 6 times more likely to develop CRC, with IBD-CRC accounting for 10%–15% of all IBD patient deaths.^10^

The risk of developing IBD-CRC has been shown to increase with disease extent and duration, while increased risk of developing sCRC is more commonly associated with certain polyp subtypes, as well as polyp size and number.^11^^,^^12^ For both IBD-CRC and sCRC, assessment of these characteristics requires patients to undergo a colonoscopy with subsequent pathological evaluation of the degree of dysplasia or polyp attributes, respectively. In both cases, pathological assessment is key to evaluating a patient’s risk of CRC, and subsequently guiding their treatment modality. Unfortunately, the difficulty in distinguishing true dysplasia from chronic colonic inflammatory changes, coupled with the development of CRC in patients without prior dysplasia, has resulted in a lack of consistency among pathologists as to the risk stratification and diagnosis of these patients.^13^ As such, there is an urgent need for novel tissue-based biomarkers in this setting, that have the potential to supplement current surveillance measures and to aid the effective pathological risk stratification of CRC tissue.

Caspases are an evolutionarily conserved family of cysteine proteases involved in the cellular signaling which regulates cell death and the innate immune response.^14^^,^^15^ Previous work from our group demonstrated a positive correlation between levels of stromal caspase-4 expression and IBD disease activity, in addition to the identification of selective caspase-4 expression in the intestinal epithelium of CRC patients.^16^ Intestinal epithelial cells in areas of normal or inflamed tissue from CRC patients lacked caspase-4 expression, while high caspase-4 levels were detected in the epithelial cells of tumor tissue.^16^ Here, we profile caspase-4 expression in different polyp subtypes, as well as in histologically defined areas of resection tissue from patients with IBD-CRC and non–IBD-associated CRC (non–IBD-CRC), in an attempt to determine the point at which caspase-4 expression is switched on during progression to CRC, and whether caspase-4 represents a novel histological biomarker to aid the identification and diagnosis of early-stage CRC.

## Methods

### Clinical Samples

#### Irish CRC cohort

St. Vincent's University Hospital Healthcare Group Research and Ethics committee granted full ethical approval to conduct this study, and written informed consent was acquired for all recruited patients in accordance with local institutional ethical guidelines. A total of 59 tissues samples (n = 41, 26 males, 15 females) were taken from the tumor site (n = 31) and adjacent normal tissue (n = 28) of patients with pathologically confirmed CRC (Table A1).

#### Northern Irish IBD-CRC cohort

Ethical approval for this cohort was obtained from the Northern Ireland Biobank (11/NI/0013, Project Ref NIB15-0180).^17^ Appropriate consent was in place for the use of samples and linked deidentified data under this approval. A total of 33 tissue samples (n = 10, 3 males, 7 females) were taken from patients with IBD-CRC at sites of normal, inflamed, dysplasia-associated lesion or mass (DALM), flat dysplasia and adenocarcinoma colonic tissue, with a further 11 tissue samples (n = 11, 7 males, 4 females) obtained from patients with non–IBD-CRC at sites of adenocarcinoma (Table A2).

#### Polyp cohort

Full ethical approval to conduct this study was grant by the St. Vincent's University Hospital Healthcare Group Research and Ethics committee. Written informed consent was obtained in accordance with local institutional ethical guidelines. A total of 57 tissue samples (n = 31; 22 males, 9 females) were taken from patients with hyperplastic (HP), sessile serrated lesion (SSL), adenoma with low-grade dysplasia (LGD), and adenoma with high-grade dysplasia (HGD) polyps (Table A3).

### Caspase-4 Immunohistochemical (IHC) Analysis

For the Northern Irish IBD-CRC and Polyp cohorts, full-face tissue sections were stained. For the Irish CRC cohort, tissue microarrays (TMAs) were constructed. Each full-face tissue section from this cohort had 3 tissue cores (0.8-mm diameter) removed for TMA construction. Immunohistochemical (IHC) staining was performed on full-face and TMA formalin-fixed, paraffin-embedded tissues to assess the expression of caspase-4 (Supplementary Materials and Methods). Staining was assessed by 2 blinded reviewers using a validated semiquantitative scoring method. All stained cells were assessed by a combined score of intensity and percentage of nuclear and cytoplasmic staining.

### CD34 (Vascular Endothelial Cell Marker) Analysis in Colorectal Polyps

IHC analysis was performed using formalin-fixed, paraffin-embedded tissues to enumerate CD34 positive blood vessels within colorectal polyps (Supplementary Materials and Methods). Vessels were counted at X400 magnification, and complete vessel structure with a lumen present was necessary to be defined as a vessel. Branching structures were counted as one; those with a break in vessel continuity were counted as 2 distinct vessels. Two blinded reviewers counted 4 microscopic fields for polyps <1 cm^2^, or 8 for polyps >1 cm^2^. Average CD34 vessel counts were used as a measure of polyp vascularity.

### Mining Online Datasets

The UCSC Xena Browser was used to collect RNAseq data for CRC tumor (n = 637) and adjacent normal (n = 51) tissue.^18^ The Kaplan-Meier plotter was used to examine the effect of caspase-4 expression on the overall survival and relapse-free survival of patients with CRC (n = 1061 and n = 1,336, respectively).^19^ Details of online datasets can be found in the Supplementary Materials and Methods.

### Statistical Analysis

Statistical analysis was carried out in GraphPad Prism (v10.1.2) and RStudio (v2021.09.0). Bar charts are expressed as the mean ± the standard error of the mean. Statistical differences were assessed using a Mann-Whitney test, Wilcoxon test or Kruskal-Wallis test with Dunn’s multiple comparisons, as appropriate. Cox proportional hazards regression analysis was performed to assess the relationship between caspase-4 expression and survival. Spearman correlations and simple linear regressions were used to assess the relationships between nonparametric variables. Corrplots were created in RStudio using packages ‘Hmisc’ (v5.1-1) and ‘corrplot’ (v0.92). Further detail on the generation and interpretation of the corrplots can be found in the Supplementary Materials and Methods. Radar charts were created by scaling the data and plotting the average value for each variable in RStudio using packages ‘fmsb’ (v0.7.5) and ‘scales’ (v1.3.0). A probability of *P* < .05 was considered statistically significant.

## Results

**Figure 1:**
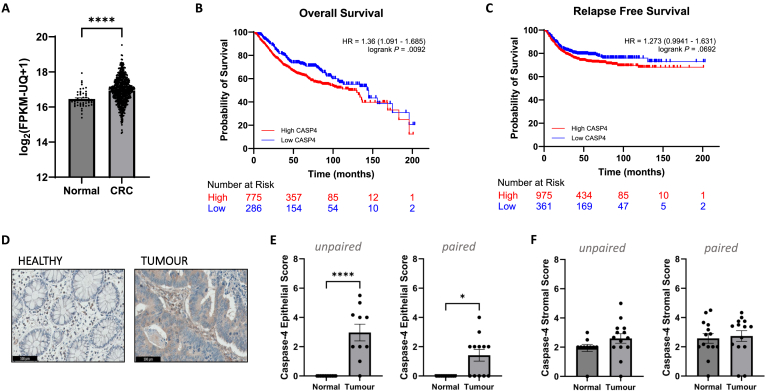
Caspase-4 expression is specifically upregulated in epithelial cells of CRC tissue. (A) RNAseq expression of CASP4 for normal (n = 51) and CRC (n = 637) tissue. Kaplan-Meier curves comparing CRC patient (B) overall survival with low (n = 286) and high (n = 775) CASP4 expression, and (C) relapse-free survival with low (n = 361) and high (n = 975) CASP4 expression. (D) Representative caspase-4 IHC staining of healthy and tumor colon tissue. (E) Tissue epithelial caspase-4 scores for unpaired adjacent normal (n = 12) vs tumor (n = 10), and paired adjacent normal (n = 12) vs tumor (n = 12). (F) Tissue stromal caspase-4 scores for unpaired adjacent normal (n = 10) vs tumor (n = 14), and paired adjacent normal (n = 14) vs tumor (n = 14) (∗*P* < .05, ∗∗∗∗*P* < .0001).

To first assess caspase-4 transcript expression in normal and CRC tissue, online databases were mined to obtain gene encoding caspase-4 (CASP4) RNAseq expression data. Significantly higher CASP4 expression was observed in CRC tissue compared to normal tissue (*P* < .0001) (Figure 1A), with no difference in CASP4 expression being detected across patient gender or age (Figure A1), or across CRC stages (*P* > .05) (Figure A2). Human guanylate-binding proteins GBP1 and GBP2, which have been shown to promote caspase-4 activation, had significant positive correlations with CASP4 in CRC tissue (*P* < .0001) (Figure A3).^20^ High CASP4 mRNA levels were associated with significantly worse overall survival in CRC patients (*P* < .01) (Figure 1B), as well as worse relapse-free survival, though not statistically significant (*P* = .069) (Figure 1C). IHC staining of patient colonic tissue (Irish CRC cohort, Table A1), revealed that caspase-4 levels were significantly increased in the epithelium of tumor compared to adjacent normal tissue. Tumor tissues that were matched to adjacent normal tissue from the same patient were analyzed as paired samples, while unmatched samples were analyzed as unpaired samples (Table A1). Both unpaired and paired samples showed significantly increased expression of epithelial caspase-4 (*P* < .0001 and *P* < .05, respectively) (Figure 1D and E). Caspase-4 was undetectable in the epithelium of all normal tissues tested. In contrast, caspase-4 was expressed in the stromal compartment of normal tissue, consistent with caspase-4 expression in infiltrating immune cell populations (Figure 1D and F).^16^

**Figure 2:**
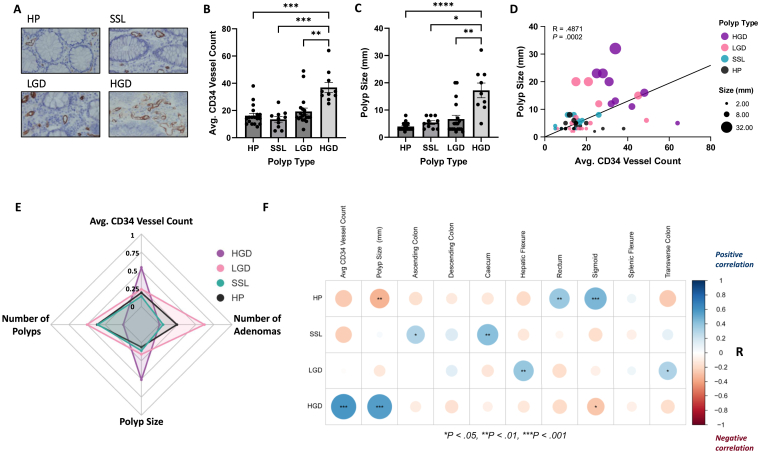
HGD are significantly larger in size and more vascular than other subtypes. (A) Representative images of CD34 IHC staining, (B) average tissue CD34 vessel counts, and (C) polyp sizes in HP (n = 18), SSL (n = 10), LGD (n = 19), and HGD (n = 10). (D) Bubble plot showing the Spearman correlation between polyp size and average tissue CD34 vessel count. (E) Radar chart showing the scaled, mean value for each variable across the polyp subtypes. (F) Corrplots depict the results of Spearman correlations between the variables labeling their corresponding row and column. Color intensity relates to R value, with positive correlations given in red and negative correlations given in blue. Circle size and interior Asterix relates to the *P* value (∗*P* < .05, ∗∗*P* < .01, ∗∗∗*P* < .001).

Having demonstrated that epithelial caspase-4 expression is elevated in CRC tissues, while absent from adjacent normal tissue, the 2 pathways of CRC development (sCRC and IBD-CRC) were investigated. To evaluate sCRC, tissue biopsies were obtained from 57 polyps of various histological subtypes (Table A2). Vascularity increased significantly from HP (*P* < .001), SSL (*P* < .001), and LGD (*P* < .01) to HGD (Figure 2A and B). Similarly, size increased from HP (*P* < .0001), SSL (*P* < .05), and LGD (*P* < .01) to HGD (Figure 2C). Vascularity and size had a significant positive correlation (*P* < .05) (Figure 2D). When the linear relationship between these 2 variables is plotted, the distinct separation between the larger LGD and HGD from the smaller HP and SSL can be observed (Figure 2D). HGD can be clearly distinguished from the other subtypes according to their vascularity and size (Figure 2E). The number of adenomas/polyps per patient did not distinguish HGD from other subtypes, with high numbers in both cases being associated with LGD (Figure 2E). HGD were shown to have significant positive correlations with vascularity (*P* < .001) and size (*P* < .001), while HP had a significant negative correlation with size (*P* < .01) (Figure 2F). Importantly, various locations along the lower gastrointestinal tract had significant correlations (either positive or negative) with polyp subtypes, indicating the subtypes most (positive) and least (negative) associated with each location (Figure 2F).

**Figure 3:**
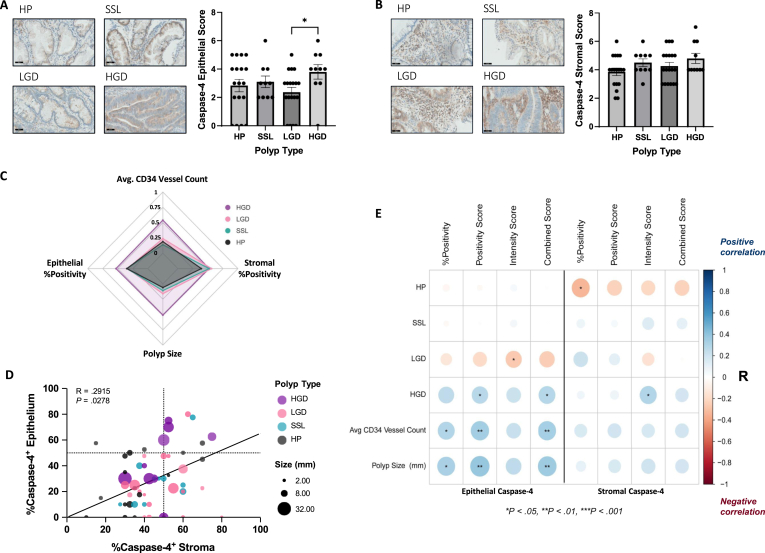
Epithelial caspase-4 is expressed in all histological polyp subtypes and expression is significantly increased upon LGD to HGD progression. Representative caspase-4 IHC staining and corresponding scores in (A) epithelial and (B) stromal cells of HP (n = 18), SSL (n = 10), LGD (n = 19), and HGD (n = 10). (C) Radar chart showing the scaled, mean value for each variable across the polyp subtypes. (D) Bubble plot showing the Spearman correlation between the percentage of caspase-4 positive epithelial and stromal tissue. (E) Corrplots depict the results of Spearman correlations between the variables labeling their corresponding row and column. Color intensity relates to R value, with positive correlations given in red and negative correlations given in blue. Circle size and interior Asterix relates to the *P* value (∗*P* < .05, ∗∗*P* < .01, ∗∗∗*P* < .001).

Next, the expression of caspase-4 in the histological subtypes of colorectal polyp (Table A2) were assessed, to determine the disease context and progression stage at which caspase-4 becomes elevated. IHC staining demonstrated that epithelial caspase-4 expression occurred in all 4 subtypes) (Figure 3A). Conventional adenomas are defined using a 2-tiered scoring system which subdivides lesions into LGD and more advanced HGD. It is therefore interesting to note that significantly increased epithelial caspase-4 expression was observed from LGD to HGD (*P* < .05) (Figure 3A). RNAseq data demonstrated no significant difference in CASP4 expression in the tissue of CRC patients with or without a colonic polyp (*P* > .05) (Figure A4), suggesting that caspase-4 expression is not dependent on the presence of a polyp. In the stroma, caspase-4 expression was evident across all polyp subtypes, consistent with an inflamed microenvironment (Figure 3B). To evaluate the performance of caspase-4 detection by IHC as a potential biomarker for risk stratification, caspase-4 epithelial and stromal positivity were compared to currently used standards for polyp risk stratification, vascularity, and size. HGD can be clearly distinguished from other subtypes using vascularity, size, and epithelial caspase-4 positivity (Figure 3C). Conversely, stromal caspase-4 positivity does not distinguish HGD from LGD and SSL. The percentage of caspase-4 positivity in the epithelium significantly correlated with that of the stromal tissue for all polyps (*P* < .05) (Figure 3D). Low percentage positivity for caspase-4 in the stromal tissue correlated significantly with HP (*P* < .05), while low epithelial intensity for caspase-4 correlated significantly with LGD (*P* < .05) (Figure 3E). Conversely, high epithelial positivity scores and combined scores, as well as high stromal intensity scores for caspase-4, were significantly elevated in HGD (*P* < .05). High epithelial caspase-4 percentage positivity, positivity score and combined score significantly correlated with vascularity (*P* < .05, *P* < .01 and *P* < .01, respectively) and size (*P* < .05, *P* < .01 and *P* < .01, respectively).

**Figure 4:**
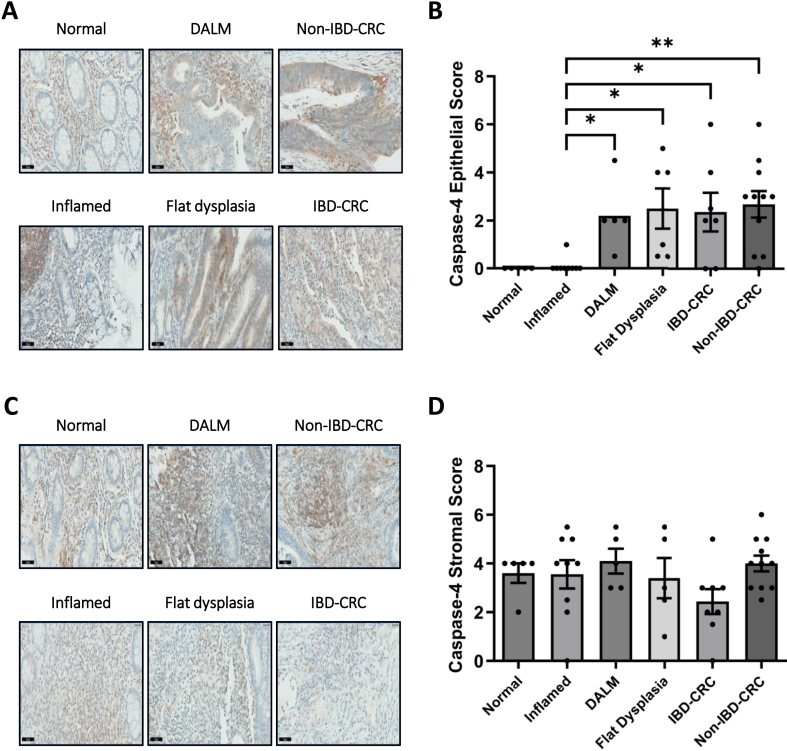
Caspase-4 epithelial expression is specifically upregulated in dysplastic and neoplastic tissue of IBD-CRC. Representative caspase-4 IHC staining and corresponding scores in the (A and B) epithelium and (C and D) stroma of histologically defined areas of normal (n = 5), inflamed (n = 9), DALM (n = 5), flat dysplasia (n = 6), IBD-CRC (n = 7), and non–IBD-CRC (n = 11) tissue (∗*P* < .05, ∗∗*P* < .01).

Having demonstrated that caspase-4 expression occurs in the tissue of all polyp subtypes, its expression profile in an IBD-CRC patient cohort was assessed (Table A3). Epithelial caspase-4 expression was absent in both adjacent normal and inflamed tissue (Figure 4A and B). However, caspase-4 expression was significantly increased in the epithelium of DALM (*P* < .05), flat dysplasia (*P* < .05), IBD-CRC (*P* < .05), and non–IBD-CRC (*P* < .01) tissue compared to inflamed tissue. Conversely, caspase-4 stromal expression was detected across all areas of IBD-CRC tissue, with no significant differences being observed (*P* > .05) (Figure 4C and D).

## Discussion

The present study demonstrates a potential role for epithelial caspase-4 as a tissue-based biomarker of both CRC risk and diagnosis. Building on our previous work,^16^ we confirm, in independent patient cohorts, that caspase-4 is selectively expressed in the epithelial compartment of CRC tumor tissue compared to adjacent normal tissue. To determine the point in CRC development when epithelial caspase-4 expression is upregulated, 2 distinct pathways of CRC development (sCRC and IBD-CRC) were interrogated. In the sCRC pathway, all histological polyp subtypes examined were found to express caspase-4 in both epithelial and stromal compartments, with significantly increased epithelial expression upon LGD to HGD progression. Differentiating high from low grade dysplasia in colorectal polyps can be challenging for histologists, therefore this result suggests that caspase-4 staining may increase the ability to distinguish a focus of HGD or T1CRC from the surrounding LGD of a polyp. However, further analysis of larger polyp cohorts is required to support this finding. For the IBD-CRC pathway, epithelial caspase-4 was specifically expressed in dysplastic and neoplastic areas of IBD-CRC tissue, but not in normal or inflamed areas. These data indicate that caspase-4 expression is upregulated or is selectively expressed in intestinal epithelial cells that have lost proliferative control, representing a promising IHC-based biomarker for CRC risk and diagnosis.

Examining the stepwise IBD-CRC pathway, caspase-4 expression presented in the epithelial cells of DALM tissue and was subsequently expressed to similar levels in the epithelium of flat dysplasia, IBD-CRC and non–IBD-CRC tissues. Progression to DALM is a worrisome step in IBD patients, with the primary aim of surveillance programs being identification of precursor dysplasia or early-stage CRC. The detection of caspase-4 in DALM epithelium could therefore provide invaluable accompaniment to current surveillance strategies.^21^ The distinction of neoplastic and chronically inflamed tissue represents a major challenge for pathologists in this setting, with current pathological markers such as p53 or ki67 demonstrating limited clinical utility, being employed as a confirmatory step rather than informative.^16^^,^^22^ Typically, 2 pathologists are required to independently confirm a diagnosis of dysplasia in IBD patients, due to the high level of interobserver variability.^23^ As such, detection of caspase-4 expression in the epithelium of colonic tissue could provide a solution to this problem.

For patients presenting with colonic polyps and progressing via the sCRC pathway, the detection of dysplastic tissue could also be aided by the addition of caspase-4 to current histopathological assessment, allowing earlier detection of high-risk individuals. While certain polyp subtypes can give an indication of CRC risk, other characteristics such as size and vascularity, are also predictive of CRC risk.^24^ In the present study, both vascularity and size are significantly increased in HGD compared to HP, SSL, and LGD, which is consistent with current risk stratification guidelines.^12^ Importantly, these characteristics correlated significantly with epithelial caspase-4 expression. Given that epithelial caspase-4 levels could also discern HGD from other subtypes, this biomarker does appear to have some utility for polyp risk stratification.

Caspase-4 and its murine ortholog caspase-11 are inflammatory enzymes, known to be involved in a number of diverse physiological functions.^25^, ^26^, ^27^, ^28^ In the context of infection, activated caspase-4 mediates noncanonical inflammasome responses in immune cells and intestinal epithelial cells, resulting in pyroptosis, a potent inflammatory form of cell death.^29^ Consistent with the inflammatory role of caspase-4, previous data have shown that stromal caspase-4 expression is associated with infiltrating macrophage, neutrophil, and lymphocyte cells, which correlates with the degree of inflammation in ulcerative colitis patient tissues.^16^ This observation fits with the growing evidence linking inflammation with polyp growth and cancer development.^30^ Previous studies have demonstrated that all polyps, regardless of their size, have a highly inflamed microenvironment. Our data support these findings, as high stromal caspase-4 expression levels, similar to levels previously observed in severely inflamed IBD tissue,^16^ were observed in all polyp subtypes examined. Expression of the inflammatory transcription factor, NFκB, has also been demonstrated to be as high in benign polyps as inflamed IBD colon tissue,^30^ supporting the involvement of inflammation early in the adenoma-carcinoma sequence. It is notable that high stromal caspase-4 levels were present in all colon tissues exhibiting epithelial positivity for caspase-4, raising the possibility that stromal inflammation above a certain threshold may be involved in driving epithelial expression of caspase-4.

## Conclusion

This study shows that caspase-4 expression occurs selectively in the intestinal epithelial cells of dysplastic and tumor tissue, and in some, but not all, polyps. The contribution of inflammasome activation and pyroptosis to cancer development is complex, depending on the cancer type, stage, tumor microenvironment and cell type.^31^ Within a chronic inflammatory environment, pyroptosis may promote tumor progression, immune evasion and metastasis; while in the context of ‘cold’ tumors, pyroptosis may serve to boost antitumor immune activity and promote cancer cell killing.^32^ Previous research from our group has identified an antitumor role for the murine ortholog caspase-11 during tumor initiation, showing that *Casp*-*11*^−/−^ mice have defective STAT1 activity, rendering them highly susceptible to the AOM-DSS model of colitis-associated cancer.^33^ Whether this observation functionally translates to human caspase-4 during CRC development is still being investigated; however, the present study shows that high caspase-4 mRNA levels in CRC patient tissue associates with worse overall survival, suggesting a protumorigenic role for caspase-4 in established CRC tumors.

Non–cell death functions of caspase-4 including cell division (via modulation of actin dynamics) and tumor angiogenesis (via Notch1 signaling regulation), have also been identified.^28^^,^^34^ These protumor functions are proposed to be independent of caspase-4 enzyme activity and occur in nonhematopoietic cells, indicating the importance of further investigation of these functions in the context of our findings. The complete absence of caspase-4 expression in the epithelium of normal colorectal tissue suggests that it may become upregulated at the initial stages of CRC development, though the mechanisms leading to this change in expression have yet to be confirmed. The differential expression profiles observed for caspase-4 in intestinal epithelial and stromal cells suggests that caspase-4 may display different functions in epithelial vs stromal compartments.

Given the poor survival rates and late-stage presentation of CRC patients, the early detection of high-risk patients is paramount. This study demonstrates that caspase-4 expression occurs in the intestinal epithelium at early stages of CRC development, representing a promising novel biomarker for CRC diagnosis.

## Supplementary Materials

Figures A1-A4

Supplementary Materials and Methods

Tables A1-A3

## References

[1] Siegel R.L., Giaquinto A.N., Jemal A. (2024). Cancer statistics, 2024. CA Cancer J Clin.

[2] Siegel R.L., Wagle N.S., Cercek A. (2023). Colorectal cancer statistics, 2023. CA Cancer J Clin.

[3] Jayasekara H., Reece J.C., Buchanan D.D. (2017). Risk factors for metachronous colorectal cancer or polyp: a systematic review and meta-analysis. J Gastroenterol Hepatol.

[4] Lu C., Schardey J., Zhang T. (2022). Survival outcomes and clinicopathological features in inflammatory bowel disease-associated colorectal cancer: a systematic review and meta-analysis. Ann Surg.

[5] Erichsen R., Baron J.A., Hamilton-Dutoit S.J. (2016). Increased risk of colorectal cancer development among patients with serrated polyps. Gastroenterology.

[6] Leggett B., Whitehall V. (2010). Role of the serrated pathway in colorectal cancer pathogenesis. Gastroenterology.

[7] Birch R.J., Burr N., Subramanian V. (2022). Inflammatory bowel disease-associated colorectal cancer epidemiology and outcomes: an English population-based study. Am J Gastroenterol.

[8] Gearhart S.L., Nathan H., Pawlik T.M. (2012). Outcomes from IBD-associated and non-IBD-associated colorectal cancer: a surveillance epidemiology and end results Medicare study. Dis Colon Rectum.

[9] Porter R.J., Arends M.J., Churchhouse A.M. (2021). Inflammatory bowel disease-associated colorectal cancer: translational risks from mechanisms to medicines. J Crohns Colitis.

[10] Mattar M.C., Lough D., Pishvaian M.J. (2011). Current management of inflammatory bowel disease and colorectal cancer. Gastrointest Cancer Res.

[11] Hassan C., Quintero E., Dumonceau J.-M. (2013). Post-polypectomy colonoscopy surveillance: European Society of Gastrointestinal Endoscopy (ESGE) guideline. Endoscopy.

[12] Hassan C., Antonelli G., Dumonceau J.-M. (2020). Post-polypectomy colonoscopy surveillance: European Society of Gastrointestinal Endoscopy (ESGE) guideline–update 2020. Endoscopy.

[13] Smits L.J., Vink-Börger E., van Lijnschoten G. (2022). Diagnostic variability in the histopathological assessment of advanced colorectal adenomas and early colorectal cancer in a screening population. Histopathology.

[14] Nicholson D. (1999). Caspase structure, proteolytic substrates, and function during apoptotic cell death. Cell Death Differ.

[15] Creagh E.M. (2014). Caspase crosstalk: integration of apoptotic and innate immune signalling pathways. Trends Immunol.

[16] Flood B., Oficjalska K., Laukens D. (2015). Altered expression of caspases-4 and-5 during inflammatory bowel disease and colorectal cancer: diagnostic and therapeutic potential. Clin Exp Immunol.

[17] Lewis C., McQuaid S., Clark P. (2018). The Northern Ireland Biobank: a cancer focused repository of science. Open J Bioresour.

[18] Goldman M.J., Craft B., Hastie M. (2020). Visualizing and interpreting cancer genomics data via the Xena platform. Nat Biotechnol.

[19] Nagy Á., Munkácsy G., Győrffy B. (2021). Pancancer survival analysis of cancer hallmark genes. Sci Rep.

[20] Dickinson M.S., Kutsch M., Sistemich L. (2023). LPS-aggregating proteins GBP1 and GBP2 are each sufficient to enhance caspase-4 activation both in cellulo and in vitro. Proc Natl Acad Sci U S A.

[21] Al Bakir I., Kabir M., Yalchin M. (2022). Optimising inflammatory bowel disease surveillance and dysplasia management—where do we stand?. United European Gastroenterol J.

[22] Yalchin M., Baker A.-M., Graham T.A. (2021). Predicting colorectal cancer occurrence in IBD. Cancers (Basel).

[23] Wijnands A.M., Mahmoud R., Lutgens M.W. (2021). Surveillance and management of colorectal dysplasia and cancer in inflammatory bowel disease: current practice and future perspectives. Eur J Intern Med.

[24] Amersi F., Agustin M., Ko C.Y. (2005). Colorectal cancer: epidemiology, risk factors, and health services. Clin Colon Rectal Surg.

[25] Li Z., Liu W., Fu J. (2021). Shigella evades pyroptosis by arginine ADP-riboxanation of caspase-11. Nature.

[26] Matikainen S., Nyman T.A., Cypryk W. (2020). Function and regulation of noncanonical caspase-4/5/11 inflammasome. J Immunol.

[27] Agnew A., Nulty C., Creagh E.M. (2021). Regulation, activation and function of caspase-11 during health and disease. Int J Mol Sci.

[28] Sumida K., Doi T., Obayashi K. (2024). Caspase-4 has a role in cell division in epithelial cells through actin depolymerization. Biochem Biophys Res Commun.

[29] Naseer N., Zhang J., Bauer R. (2022). Salmonella enterica serovar typhimurium induces NAIP/NLRC4-and NLRP3/ASC-independent, caspase-4-dependent inflammasome activation in human intestinal epithelial cells. Infect Immun.

[30] Berkovich L., Gerber M., Katzav A. (2022). NF-kappa B expression in resected specimen of colonic cancer is higher compared to its expression in inflammatory bowel diseases and polyps. Sci Rep.

[31] Xia X., Wang X., Cheng Z. (2019). The role of pyroptosis in cancer: pro-cancer or pro-“host”?. Cell Death Dis.

[32] Zhivaki D., Kagan J.C. (2021). NLRP3 inflammasomes that induce antitumor immunity. Trends Immunol.

[33] Flood B., Manils J., Nulty C. (2019). Caspase-11 regulates the tumour suppressor function of STAT1 in a murine model of colitis-associated carcinogenesis. Oncogene.

[34] Fan L., Liu H., Zhu G. (2022). Caspase-4/11 is critical for angiogenesis by repressing Notch1 signalling via inhibiting γ-secretase activity. Br J Pharmacol.

