# Supplementary material for: Caspase-4 Has Potential Utility as a Colorectal Tissue Biomarker for Dysplasia and Early-Stage Cancer
Source: Gastro Hep Adv. 2024 Sep 16;4(2):100552. doi: 10.1016/j.gastha.2024.09.007 (PMC11760840; doi:10.1016/j.gastha.2024.09.007)
Supplement: Figures A1-A4 [file mmc1.docx]

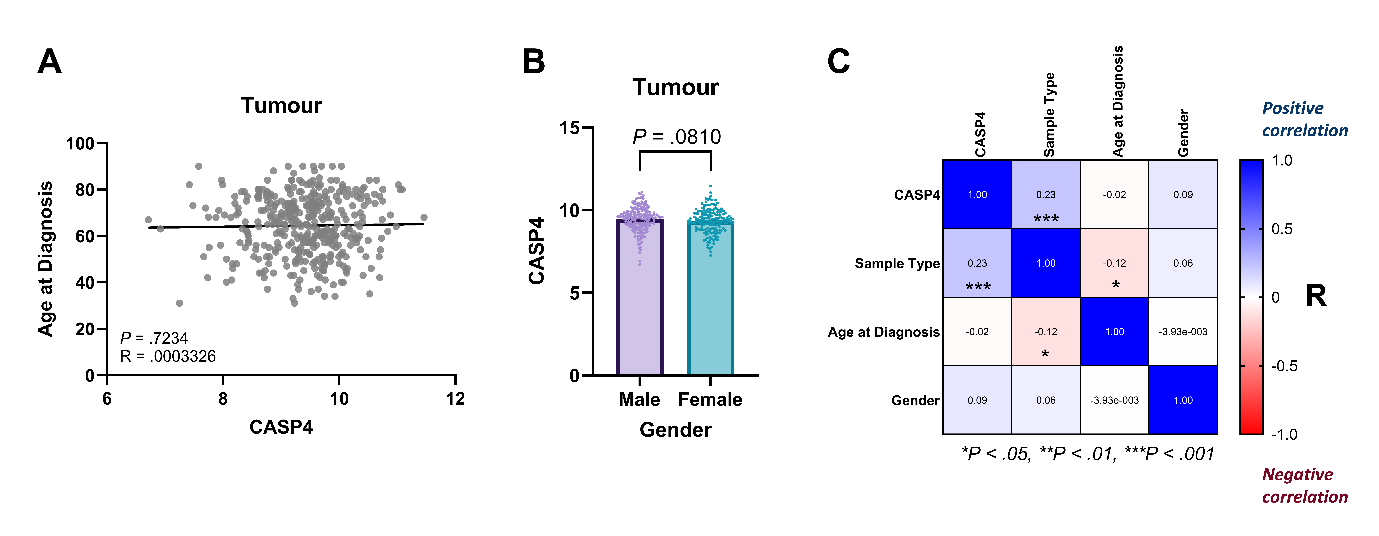


**Supplementary Figure S1. Caspase-4 expression does not correlate with age and is not significantly different between genders**. (**A**) Spearman correlation of RNAseq expression of CASP4 in Log_2_(FPKM-UQ+1) in CRC tissue with patient age at diagnosis (n=379). (**B**) RNAseq expression of CASP4 in Log_2_(FPKM-UQ+1) in CRC tissue for male (n=208) and female (n=171) patients. (**C**) Correlation matrix showing multivariate analysis of RNAseq expression of CASP4 in Log_2_(FPKM-UQ+1) with patient clinical characteristics. Spearman correlation R values are given in each cell. Cell colours demonstrate positive (blue) and negative (red) corelations. Categorical variables (Sample Type, Gender) are binary coded. For Sample Type, (Normal=0, Tumour=1) positive correlations indicate association with CRC tissue and negative correlations indicate association with Normal tissue. For Gender (Female=0, Male=1) positive correlations indicate association with Males and negative correlations indicate association with Females. (**P* < .05, ***P* < .01, ****P* < .001).


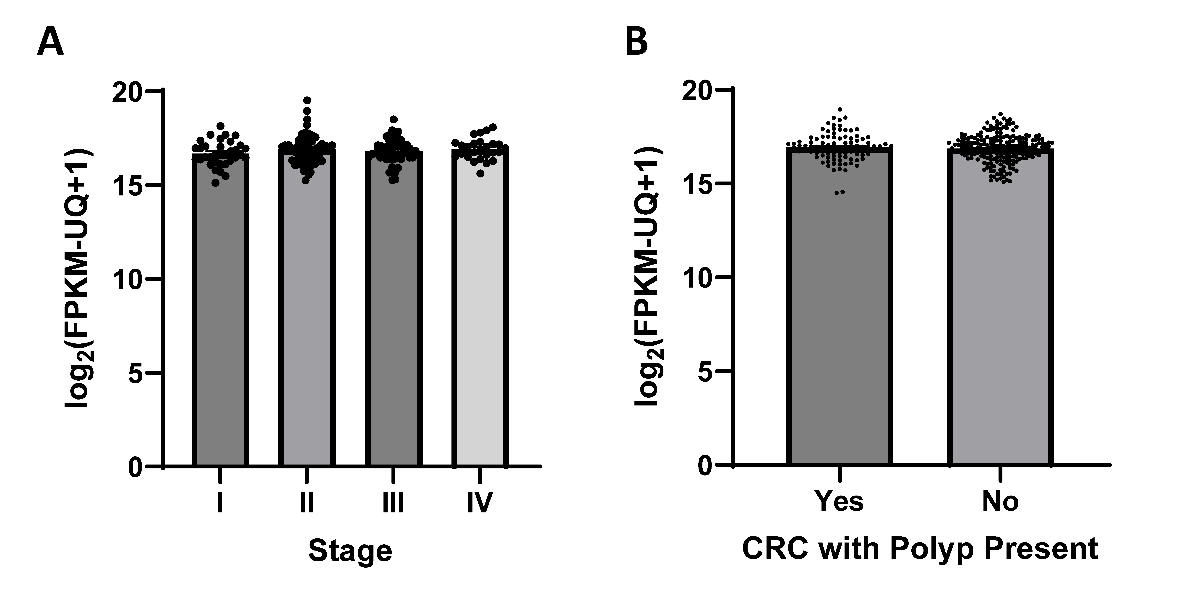


**Supplementary Figure S2.** **Caspase-4 expression is not dependent on cancer stage**. RNAseq expression of CASP4 in Log_2_(FPKM-UQ+1) for stage I (n=34), stage II (n=53), stage III (n=53), and stage IV (n=26) CRC tissue.


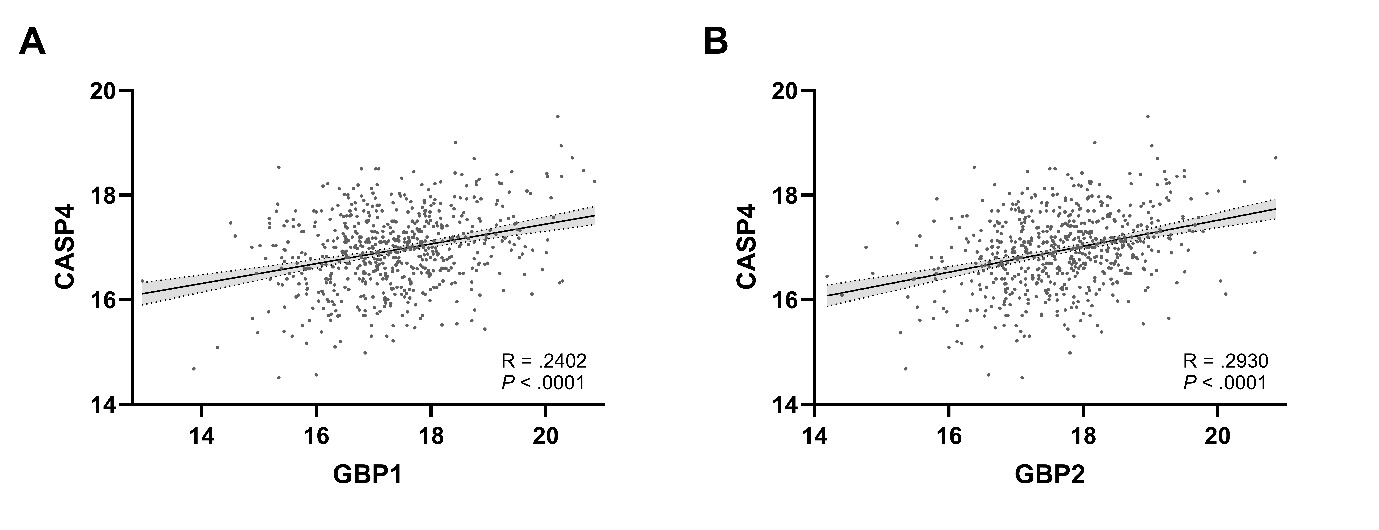


**Supplementary Figure S3. Caspase-4 expression has a significant positive correlation with GBP1 and GBP2 expression**. RNAseq expression of CASP4 against (**A**) GBP1 and (**B**) GBP2 in Log_2_(FPKM-UQ+1) for primary CRC tissue (n=635).


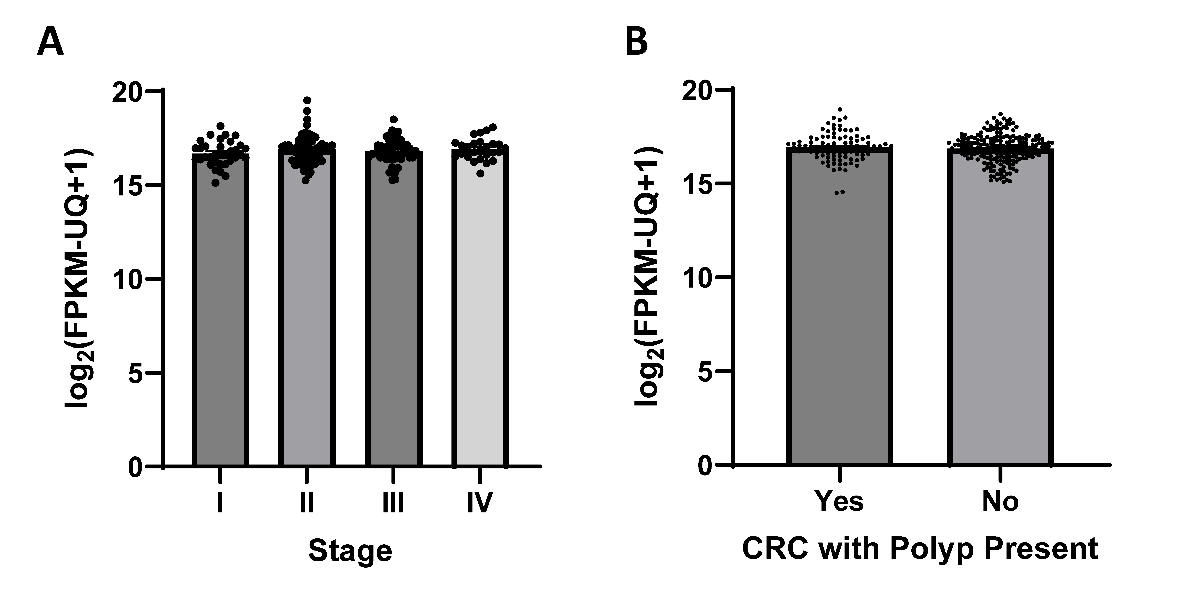


**Supplementary Figure S4. Caspase-4 expression is not dependent polyp presence**. RNAseq expression of CASP4 in Log_2_(FPKM-UQ+1) for CRC tissue of patients with (n=96) and without (n=218) a colonic polyp.
