# Supplementary material for: Caspase-4 Has Potential Utility as a Colorectal Tissue Biomarker for Dysplasia and Early-Stage Cancer
Source: Gastro Hep Adv. 2024 Sep 16;4(2):100552. doi: 10.1016/j.gastha.2024.09.007 (PMC11760840; doi:10.1016/j.gastha.2024.09.007)
Supplement: Supplementary Materials and Methods [file mmc2.docx]

**Supplementary Materials and Methods**

***Caspase-4 IHC analysis of colorectal tissue***

Immunohistochemistry of tissue samples was performed using formalin-fixed, paraffin-embedded (FFPE) tissues and TMAs (for the Irish CRC cohort) to assess the expression of caspase-4. A three-stage immunoperoxidase labelling technique incorporating avidin–biotin immunoperoxidase complex (Dako, Glostrup, Denmark) was carried out. Tissue sections were incubated with primary anti-caspase-4 (Medical and Biological Laboratories (MBL), Woburn,MA, USA) antibody for 1 h. Sections were also incubated with an appropriate isotype-matched mouse/rabbit monoclonal antibody as a negative control. Diaminobenzadine tetrahydrochloride (DAB; Sigma) was used to visualize staining. Images were captured using the Olympus DP50 light microscope and AnalySIS software (Soft Imaging System Corporation, Lakewood, CO, USA). Staining intensity was graded using a scale of 0–3 (0=negative, 1=weak, 2=moderate, 3=strong), and percentage positivity was graded using a scale of 0–4 (0=0%, 1=1–24%, 2=25–49%, 3=50–74%, 4=75–100%). For the TMAs, tissue cores were stained in triplicate for each tumour/adjacent normal tissue. The average from the triplicate scores was calculated and used to determine the final IHC score. Tissues that did not have triplicate cores stained were excluded from the analysis.

***Caspase-4 IHC analysis of colorectal polyps***

IHC of polyp tissue samples was performed using FFPE tissues to assess the expression of caspase-4 on a Bond-III immunostainer from Leica Biosystems (Newcastle upon Tyne, UK). The Bond-III system dewaxed slides prior to pre-treatment with Bond Epitope Retrieval Solution I. Primary anti-Casp-4 (MBL) (Santa Cruz Biotechnologies Inc., Dallas, TX, USA) antibody was diluted in Bond primary antibody diluent. Detection and visualization of stained cells was achieved using the Bond Polymer Refine Detection Kit, using DAB as the chromagen. Tissues were counterstained with haematoxylin and coverslipped. Appropriate negative controls (omission of primary antibodies) were used in all assays. Staining intensity was graded using a scale of 0–3 (0=negative, 1=weak, 2=moderate, 3=strong), and percentage positivity was graded using a scale of 0–4 (0=0%, 1=1–24%, 2=25–49%, 3=50–74%, 4=75–100%).

***CD34 immunohistochemical analysis of colorectal polyps***

Immunohistochemistry of tissue samples was performed using formalin-fixed, paraffin-embedded (FFPE) tissues to enumerate CD34 positive blood vessels within colorectal polyps. Staining was carried out on the Ventana Benchmark GX semi-automated immunostainer (Roche Tissue Diagnostics, Oro Valley, AZ, USA). The Benchmark system dewaxed the slides prior to pre-treatment with a standard cell conditioning solution for 1h. Tissue sections were incubated with primary anti-CD34 (Company) antibody for 1h. Sections were also incubated with an appropriate isotype-matched mouse/rabbit monoclonal antibody as a negative control. Diaminobenzadine tetrahydrochloride (DAB; Sigma) was used to visualize staining, with haematoxylin as the counterstain.

***Mining online datasets***

The UCSC Xena Browser was utilised to collect RNAseq data for CRC tumour (n=637) and adjacent-normal (n=51) tissue^1^. TCGA samples with primary colon cancer (GDC-TCGA-COAD; n=470) and primary rectal cancer (GDC-TCGA-READ; n=167) were both included (dbGaP Study Accession: phs000178). Caspase-4, GBP1 and GBP2 expression levels were exported as log_2_(FPKM-UQ+1), alongside relevant clinical information.

The Kaplan-Meier plotter was used to examine the effect of low and high caspase-4 expression on the overall survival and relapse-free survival of patients with CRC (n=1,061 and n=1,336, respectively). The following datasets were included in this analysis of overall survival: GSE12945 (n=62), GSE17538 (n=232), GSE29621 (n=8), GSE38832 (n=70), GSE39582 (n=505), GSE41258 (n=184). Relapse-free survival was assessed using the following datasets: GSE12945 (n=62), GSE14333 (n=123), GSE143985 (n=91), GSE17538 (n=164), GSE29621 (n=8), GSE31595 (n=20), GSE33114 (n=90), GSE37892 (n=65), GSE38832 (n=70), GSE39582 (n=469), GSE41258 (n=115), GSE92921 (n=59).

**Corrplots**

Corrplots were created in RStudio using packages ‘Hmisc’ (v5.1-1) and ‘corrplot’ (v0.92). Corrplots depict the results of Spearman correlations between the variables labelling their corresponding row and column. Continuous variables are unchanged, while categorical variables such as Polyp Type, are binary coded as 0=Absent and 1=Present. Positive correlations, indicated by positive R values, are given in red, while negative correlations, indicated by negative R values, are given in blue. Significant correlations are indicated by Asterix according to their level of significance (**P* < .05, ***P* < .01, ****P* < .001). A representative line graph of the Spearman correlation between the various Polyp Types and Average CD34 Vessel Count is given below to aid interpretation (**Supplementary Materials and Methods Figure S1**).


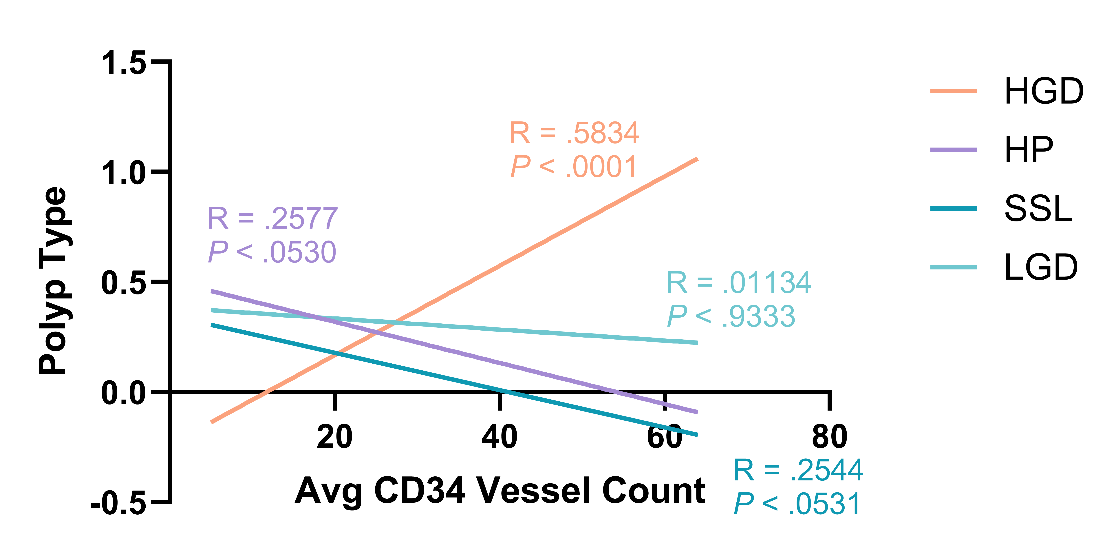


**Supplementary Materials and Methods Figure S1.** Representative line graph of the Spearman correlation between Polyp Type and Average CD34 Vessel Count (this graph relates to the first column of the Corrplot in Figure 2F).

1. Goldman MJ, Craft B, Hastie M, et al. Visualizing and interpreting cancer genomics data via the Xena platform. Nat Biotechnol. 2020;38(6):675-8.
