# Supplementary material for: Caspase-4 Has Potential Utility as a Colorectal Tissue Biomarker for Dysplasia and Early-Stage Cancer
Source: Gastro Hep Adv. 2024 Sep 16;4(2):100552. doi: 10.1016/j.gastha.2024.09.007 (PMC11760840; doi:10.1016/j.gastha.2024.09.007)
Supplement: Tables A1-A3 [file mmc3.docx]

**Supplementary Table S1. Clinical characteristics of the Irish CRC cohort.**

| *Cohort details* | | |  |  | |  |  |
| --- | --- | --- | --- | --- | --- | --- | --- |
|  |  |  | **Normal** | | **Tumour** |  |  |
| **Number of Patients (M/F)** | | | 28 (17/11) | | 31 (17/14) |  |  |
| **Number of Unpaired (M/F)** | | | 10 (9/1) | | 13 (9/4) |  |  |
| **Number of Paired (M/F)** | | | 18 (8/10) | | 18 (8/10) |  |  |
| **Average Age (Range)** | | | 72 (31-84) | | 73 (31-84) |  |  |
|  |  |  |  | |  |  |  |
| *Individual case details* | | |  |  | |  |  |
| **Patient #** | **Gender** | **Age** | **Normal** | | **Tumour** | **Site of Surgery** | **TNM Stage** |
| **1** | Female | 69 | x | | x | Sigmoid Colon | pT3N0Mx |
| **2** | Female | 80 | x | | x | Colon | pT4N0MxR0 |
| **3** | Male | 82 | x | |  | Colon | pT4N0M0 |
| **4** | Male | 57 | x | |  | Caecum | pT3N0Mx |
| **5** | Male | 66 |  | | x | Colon | pT3N0MxR0 |
| **6** | Male | 71 | x | |  | Rectum | PT3N0Mx |
| **7** | Male | 83 | x | |  | Sigmoid Colon | pT3N0M0 |
| **8** | Male | 70 | x | |  | Colon | pT3N0M0 |
| **9** | Male | 70 | x | | x | Colon | pT4N0R0 |
| **10** | Male | 62 | x | |  | Rectum | pT3N0Mx |
| **11** | Female | 76 |  | | x | Sigmoid Colon/Rectum | pT3N0MxR0 |
| **12** | Male | 68 | x | | x | Rectum | pT3N0Mx |
| **13** | Male | 67 |  | | x | Transverse Colon | pT4N0Mx |
| **14** | Female | 81 | x | | x | Splenic Flexure | pT3N0M0 |
| **15** | Male | 80 |  | | x | Sigmoid Colon | pT4N0R0 |
| **16** | Female | 84 | x | | x | Colon | pT3N0MxR0 |
| **17** | Male | 76 | x | | x | Sigmoid Colon | pT4N0R0 |
| **18** | Male | 80 |  | | x | Transverse Colon | pT3N0M0 |
| **19** | Female | 75 | x | | x | Caecum | pT3N0M0 |
| **20** | Female | 84 | x | | x | Rectum | pT3N0MxR0 |
| **21** | Male | 81 |  | | x | Colon | pT3N0MxR0 |
| **22** | Female | 67 | x | | x | Sigmoid Colon | pT3N0 |
| **23** | Male | 71 | x | |  | Rectum | pT3N0Mx |
| **24** | Female | 76 |  | | x | Colon | pT4N0MxR0 |
| **25** | Male | 72 | x | | x | Colon | PT3N0MxR0 |
| **26** | Male | 79 | x | | x | Rectum | pT3N0MxR0 |
| **27** | Female | 56 | x | | x | Sigmoid Colon | pT3N0M0 |
| **28** | Female | 69 |  | | x | Colon | pT3N0MxR0 |
| **29** | Male | 74 | x | | x | Transverse Colon | pT3N0 |
| **30** | Male | 84 | x | |  | Sigmoid Colon | pT3N0Mx |
| **31** | Female | 77 | x | |  | Sigmoid Colon | pT3N1Mx |
| **32** | Female | 79 | x | | x | Rectum | pT3N0Mx |
| **33** | Male | 73 | x | | x | Sigmoid Colon | pT4N0MxR0 |
| **34** | Female | 31 | x | | x | Rectum | PT3N0MxR0 |
| **35** | Male | 76 | x | |  | Rectum | pT3N0MxR1 |
| **36** | Male | 76 |  | | x | Sigmoid Colon/Rectum | pT3N0Mx |
| **37** | Female | 57 |  | | x | Sigmoid Colon | pT4N0Mx |
| **38** | Male | 75 |  | | x | Colon | pT3N0M0 |
| **39** | Male | 73 |  | | x | Sigmoid Colon | pT3N0MxR0 |
| **40** | Male | 77 |  | | x | Colon | pT3N0MxR0 |
| **41** | Male | 77 | x | | x | Sigmoid Colon | pT3N0Mx |

Age is given in years. Average age is rounded to the nearest whole number.

**Supplementary Table S2. Clinical characteristics of the Northern Irish IBD-CRC cohort.**

| *Cohort details* | | |  | |  | |  | |  | |  | |  | |  |  | |  |
| --- | --- | --- | --- | --- | --- | --- | --- | --- | --- | --- | --- | --- | --- | --- | --- | --- | --- | --- |
|  |  |  | **Normal** | **Inflamed** | | **DALM** | | **Flat dysplasia** | | **IBD-CRC** | | **Non-IBD-CRC** | |  | | |  | |
| **Number of samples (M/F)** | | | 5 (3/2) | 9 (6/3) | | 5 (3/2) | | 6 (5/1) | | 8 (6/2) | | 11 (7/4) | |  | | |  | |
| **Average Age (Range)** | | | 60 (49-77) | 60 (33-77) | | 61 (33-77) | | 56 (33-65) | | 65 (54-77) | | 67 (51-77) | |  | | |  | |
|  |  |  |  |  | |  | |  | |  | |  | |  | | |  | |
| *Individual case details* | | |  | |  | |  | |  | |  | |  | |  |  | |  |
| **Case #** | **Gender** | **Age** | **Normal** | **Inflamed** | | **DALM** | | **Flat dysplasia** | | **IBD-CRC** | | **Non-IBD-CRC** | | **Site of surgery** | | | **Most advanced diagnosis** | |
| 1 | Female | 33 |  | x | | x | | x | |  | |  | | Rectum/Anus | | | Flat dysplasia | |
| 2 | Male | 49 | x | x | |  | | x | |  | |  | | Colon | | | Flat dysplasia | |
| 3 | Female | 77 | x | x | | x | |  | | x | |  | | Sigmoid Colon | | | Adenocarcinoma | |
| 4 | Male | 65 |  | x | | x | | x | | x | |  | | Rectum | | | Adenocarcinoma | |
| 5 | Male | 64 |  | x | | x | | x | | x | |  | | Colon | | | Adenocarcinoma | |
| 6 | Male | 64 |  | x | | x | | x | | x | |  | | Rectum | | | Adenocarcinoma | |
| 7 | Male | 58 | x | x | |  | | x | | x | |  | | Colon | | | Adenocarcinoma | |
| 8 | Male | 63 | x |  | |  | |  | | x | |  | | Rectum | | | Adenocarcinoma | |
| 9 | Female | 54 | x | x | |  | |  | | x | |  | | Rectum | | | Adenocarcinoma | |
| 10 | Male | 74 |  | x | |  | |  | | x | |  | | Colon/Rectum/Anus | | | Adenocarcinoma | |
| 11 | Female | 72 |  |  | |  | |  | |  | | x | | Sigmoid Colon | | | Adenocarcinoma | |
| 12 | Male | 64 |  |  | |  | |  | |  | | x | | Sigmoid Colon | | | Adenocarcinoma | |
| 13 | Male | 66 |  |  | |  | |  | |  | | x | | Sigmoid Colon | | | Adenocarcinoma | |
| 14 | Female | 77 |  |  | |  | |  | |  | | x | | Sigmoid Colon | | | Adenocarcinoma | |
| 15 | Female | 75 |  |  | |  | |  | |  | | x | | Rectum | | | Adenocarcinoma | |
| 16 | Male | 57 |  |  | |  | |  | |  | | x | | Sigmoid Colon | | | Adenocarcinoma | |
| 17 | Male | 60 |  |  | |  | |  | |  | | x | | Sigmoid Colon | | | Adenocarcinoma | |
| 18 | Male | 79 |  |  | |  | |  | |  | | x | | Sigmoid Colon | | | Adenocarcinoma | |
| 19 | Female | 66 |  |  | |  | |  | |  | | x | | Sigmoid Colon/Rectum | | | Adenocarcinoma | |
| 20 | Male | 69 |  |  | |  | |  | |  | | x | | Sigmoid Colon/Rectum | | | Adenocarcinoma | |
| 21 | Male | 51 |  |  | |  | |  | |  | | x | | Sigmoid Colon | | | Adenocarcinoma | |

Age is given in years. Average age is rounded to the nearest whole number. DALM – dysplasia-associated lesion or mass; IBD-CRC – inflammatory bowel disease-associated colorectal cancer; non-IBD-CRC – non-inflammatory bowel disease-associated colorectal cancer.

**Supplementary Table S3. Clinical characteristics of the polyp cohort.**

| *Cohort details* | | |  | |  | |  | |  | |  | |  |  | |  |
| --- | --- | --- | --- | --- | --- | --- | --- | --- | --- | --- | --- | --- | --- | --- | --- | --- |
|  |  |  | **HP** | **SSL** | | **LGD** | | **HGD** | |  | |  | | |  | |
| **Number of Polyps (M/F)** | | | 18 (11/7) | 10 (9/1) | | 19 (19/0) | | 10 (6/4) | |  | |  | | |  | |
| **Average Age (Range)** | | | 69 (61-70) | 69 (68-70) | | 70 (68-70) | | 67 (62-70) | |  | |  | | |  | |
| **Average Polyp Size** | | | 3.9mm | 5.6mm | | 6.2mm | | 17.2mm | |  | |  | | |  | |
| *Individual case details* | | |  | |  | |  | |  | |  | |  |  | |  |
| **Patient #** | **Gender** | **Age** | **HP** | **SSL** | | **LGD** | | **HGD** | | **Size** | | **Site of Polyp** | | | **Polyp** # | |
| **1** | Male | 70 |  |  | | x | |  | | 5 | | Hepatic Flexure | | | 1 | |
|  |  |  |  |  | | x | |  | | 7 | | Transverse Colon | | | 2 | |
|  |  |  | x |  | |  | |  | | 5 | | Splenic Flexure | | | 3 | |
|  |  |  |  |  | | x | |  | | 3 | | Descending Colon | | | 4 | |
|  |  |  |  |  | | x | |  | | 20 | | Sigmoid Colon | | | 5 | |
|  |  |  | x |  | |  | |  | | 5 | | Rectum | | | 6 | |
| **2** | Male | 70 |  |  | | x | |  | | N/A | | Ascending Colon | | | 7 | |
|  |  |  | x |  | |  | |  | | 3 | | Sigmoid Colon | | | 8 | |
|  |  |  | x |  | |  | |  | | 3 | | Rectum | | | 9 | |
|  |  |  | x |  | |  | |  | | 3 | | Rectum | | | 10 | |
|  |  |  |  |  | | x | |  | | 3 | | Rectum | | | 11 | |
| **3** | Male | 70 |  | x | |  | |  | | 4 | | Descending Colon | | | 12 | |
|  |  |  |  |  | | x | |  | | 15 | | Sigmoid Colon | | | 13 | |
|  |  |  |  | x | |  | |  | | 6 | | Rectum | | | 14 | |
| **4** | Male | 69 |  |  | | x | |  | | 3 | | Transverse Colon | | | 15 | |
|  |  |  |  |  | | x | |  | | 3 | | Transverse Colon | | | 16 | |
|  |  |  |  |  | | x | |  | | 5 | | Descending Colon | | | 17 | |
| **5** | Male | 70 |  |  | | x | |  | | 3 | | Hepatic Flexure | | | 18 | |
|  |  |  |  |  | | x | |  | | 6 | | Sigmoid Colon | | | 19 | |
|  |  |  | x |  | |  | |  | | 3 | | Rectum | | | 20 | |
| **6** | Male | 69 |  | x | |  | |  | | 8 | | Caecum | | | 21 | |
|  |  |  |  | x | |  | |  | | 5 | | Transverse Colon | | | 22 | |
|  |  |  |  | x | |  | |  | | 3 | | Descending Colon | | | 23 | |
| **7** | Male | 68 |  | x | |  | |  | | 8 | | Ascending Colon | | | 24 | |
|  |  |  |  | x | |  | |  | | 3 | | Sigmoid Colon | | | 25 | |
|  |  |  | x |  | |  | |  | | 8 | | Sigmoid Colon | | | 26 | |
| **Patient #** | **Gender** | **Age** | **HP** | **SSL** | | **LGD** | | **HGD** | | **Size** | | **Site of Polyp** | | | **Polyp** # | |
| **8** | Male | 68 |  | x | |  | |  | | 3 | | Transverse Colon | | | 27 | |
|  |  |  | x |  | |  | |  | | 3 | | Sigmoid Colon | | | 28 | |
| **9** | Male | 70 |  |  | | x | |  | | 20 | | Sigmoid Colon | | | 29 | |
|  |  |  |  |  | | x | |  | | 2 | | Hepatic Flexure | | | 30 | |
| **10** | Male | 68 |  |  | | x | |  | | 3 | | Sigmoid Colon | | | 31 | |
|  |  |  | x |  | |  | |  | | 5 | | Descending Colon | | | 32 | |
| **11** | Male | 70 |  |  | | x | |  | | 3 | | Hepatic Flexure/Descending Colon | | | 33 | |
|  |  |  |  |  | | x | |  | | 3 | | Transverse Colon | | | 34 | |
| **12** | Male | 68 |  |  | | x | |  | | 2 | | Transverse Colon | | | 35 | |
|  |  |  |  |  | | x | |  | | 12 | | Splenic Flexure | | | 36 | |
| **13** | Male | 68 |  | x | |  | |  | | 8 | | Caecum | | | 37 | |
|  |  |  | x |  | |  | |  | | 6 | | Sigmoid Colon | | | 38 | |
| **14** | Female | 68 |  | x | |  | |  | | 6 | | Ascending Colon | | | 39 | |
|  |  |  | x |  | |  | |  | | 5 | | Descending Colon | | | 40 | |
| **15** | Female | 69 | x |  | |  | |  | | 3 | | Sigmoid Colon | | | 41 | |
| **16** | Male | 69 | x |  | |  | |  | | 3 | | Rectum | | | 42 | |
| **17** | Female | 68 | x |  | |  | |  | | 4 | | Sigmoid Colon | | | 43 | |
| **18** | Female | 61 | x |  | |  | |  | | 4 | | Sigmoid Colon | | | 44 | |
| **19** | Male | 68 | x |  | |  | |  | | 2 | | Rectum | | | 45 | |
| **20** | Male | 70 | x |  | |  | |  | | 3 | | Sigmoid Colon | | | 46 | |
| **21** | Female | 68 | x |  | |  | |  | | 5 | | Sigmoid Colon | | | 47 | |
| **22** | Male | 70 |  |  | |  | | x | | 5 | | Descending Colon | | | 48 | |
| **23** | Male | 67 |  |  | |  | | x | | 12 | | Sigmoid Colon | | | 49 | |
| **24** | Female | 62 |  |  | |  | | x | | 32 | | Sigmoid Colon | | | 50 | |
| **25** | Female | 67 |  |  | |  | | x | | 23 | | Sigmoid Colon | | | 51 | |
| **26** | Male | 67 |  |  | |  | | x | | 13 | | Sigmoid Colon/Rectum | | | 52 | |
| **27** | Female | 66 |  |  | |  | | x | | 16 | | Rectum | | | 53 | |
| **28** | Male | 65 |  |  | |  | | x | | 11 | | Sigmoid Colon | | | 54 | |
| **29** | Female | 65 |  |  | |  | | x | | N/A | | Sigmoid Colon | | | 55 | |
| **30** | Male | 69 |  |  | |  | | x | | 23 | | Rectum | | | 56 | |
| **31** | Male | 68 |  |  | |  | | x | | 20 | | Sigmoid Colon | | | 57 | |

Age is given in years. Average age is rounded to the nearest whole number. Polyp size is given in mm. HGD – high-grade dysplasia; HP – hyperplastic; LGD – low-grade dysplasia; SSL – sessile serrated lesion
